# Cardiac disease risk prediction using machine learning algorithms

**DOI:** 10.1049/htl2.12053

**Published:** 2023-11-30

**Authors:** Albert Alexander Stonier, Rakesh Krishna Gorantla, K Manoj

**Affiliations:** ^1^ Department of Energy and Power Electronics, School of Electrical Engineering Vellore Institute of Technology Vellore India; ^2^ Department of Control and Automation, School of Electrical Engineering Vellore Institute of Technology Vellore India

**Keywords:** cardiac disease, decision tree, heart attack, KNN, machine learning, naive Bayes, neural networks, prediction, random forest, regression models, support vector machine

## Abstract

Heart attack is a life‐threatening condition which is mostly caused due to coronary disease resulting in death in human beings. Detecting the risk of heart diseases is one of the most important problems in medical science that can be prevented and treated with early detection and appropriate medical management; it can also help to predict a large number of medical needs and reduce expenses for treatment. Predicting the occurrence of heart diseases by machine learning (ML) algorithms has become significant work in healthcare industry. This study aims to create a such system that is used for predicting whether a patient is likely to develop heart attacks, by analysing various data sources including electronic health records and clinical diagnosis reports from hospital clinics. ML is used as a process in which computers learn from data in order to make predictions about new datasets. The algorithms created for predictive data analysis are often used for commercial purposes. This paper presents an overview to forecast the likelihood of a heart attack for which many ML methodologies and techniques are applied. In order to improve medical diagnosis, the paper compares various algorithms such as Random Forest, Regression models, K‐nearest neighbour imputation (KNN), Naïve Bayes algorithm etc. It is found that the Random Forest algorithm provides a better accuracy of 88.52% in forecasting heart attack risk, which could herald a revolution in the diagnosis and treatment of cardiovascular illnesses.

## Graphical abstract

1. Prediction of cardiac severity with aid of machine learning algorithms

2. Comparative analysis of various machine learning algorithms

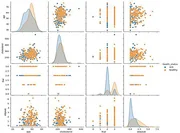

## 1 INTRODUCTION

Myocardial infarction (MI), which is also called heart attack, occurs when a clot of plasma prevents the circulation of blood to a portion of the heart. This blockage can hurt the heart muscle and can lead to complications that are life‐threatening. Cholesterol, age, gender, smoking, genes, BP (blood‐pressure), and being overweight are the causes of heart attack. Heart attack risk prediction is a person's best guess of how likely it is that they will have a heart attack or MI in the future. Nowadays, researchers are focusing mostly on detecting the prediction of the heart problem because predicting with more accuracy is a quite tedious process. If the prediction is not accurate, it will lead to huge mortality rate increase irrespective of any age. Inducing machine learning (ML) to predict risk in heart disease was developed, but the problem persists in the elimination of complex associations in‐between heart function and risk prediction. For the different regions of population, the predictor is not predicted accurately. Identifying heart health problem much earlier will allow us to treat the patient quite easily and it will minimize the mortality rate of the infected population and minimize cost for treating patients.

ML can predict the chance of a cardiac risk in a more personalized way that can improve the accuracy of risk assessment. ML algorithms can look at a lot of different types of data, like medical records, life‐style data, and genetics, to find trends and make very accurate predictions. In addition, other sets of data such as demographic, clinical, and genetic data are collected and studied to find out what makes someone more likely to have a heart attack. The algorithm then uses this knowledge to make a model that can predict how likely it is that a person will have a heart attack. ML algorithms can also find new risk factors that may not be obvious with traditional risk assessment methods. For example, ML algorithms can look at a lot of data to find connections between lifestyle factors and the chance of a heart attack that might not be obvious at first glance.

The proposed model anticipated must be efficient when compared to the existing model. Training a learning model to perform various automating data analytics is known as ML, and it is frequently employed in medical research. The statistical techniques might not be able to execute in certain arena, while ML can be implemented on complex data, such as biological and medical data [4].

## 2 LITERATURE SURVEY

Paranthaman et al. [1] used deep learning to predict cardiovascular disease. They discussed various intelligent techniques such as neural networks, perceptron algorithms, and so on to detect the disease and also to differentiate the various problems that arise while predicting. The authors also used the various ML techniques that involve single‐layered neural networks and multilayer neural networks for classifying the problem.

Deepika and Balaji [2] worked on the Multilayer Perceptron Algorithm in which the pre‐processed heart disease datasets are used as input in the proposed effort. Following pre‐processing, the optimized unsupervised technique for feature selection is used. Considering the chosen features the multilayer perceptron‐Enhanced Brownian Motion based on Dragonfly Algorithm classifier was used to forecast human heart disease. Finally, the effectiveness of the suggested process has been evaluated on several factors.

Xie et al. [3] proposed the cardiovascular disease prediction by the weight learning‐based technique using density information. For predicting, missing data is one of the constraints for K‐nearest neighbour imputation (KNNI).

Faizal et al. [4] proposed the heart disease prediction model using the conventional and AI‐based approaches. The traditional way is still useful in predicting heart disease using the logic of linear regression and then logistic regression. It is used for many purposes and normally obtained from median and hence it is not applicable to all the patients. The ML algorithm involves three types of learning such as supervised which learns humans labelled model, unsupervised to learn hidden features without any feedback and reinforcement learning involves rewards. This reward continuously involves optimization of the heart disease prediction.

Janaranjani et al. [5] explored the study of the potential of ML algorithms in predicting heart disease and highlighted the importance of early detection and prevention of heart attacks. The authors also discussed the various exploratory methods for extracting the features of data. Finally, they concluded that the prediction of disease plays a vital role in the medical field to diagnose the patient before they are hospitalized.

Azmi et al. [6] examined the use of ML approaches in predicting cardiovascular illness in their detailed study report. They conducted a systematic review of various ML algorithms, such as decision trees, neural networks, support vector machines (SVM) and utilised in cardiovascular disease prediction. The article covers the problems of using medical big data for ML and discusses some of the advantages and limits of these techniques. Furthermore, the authors indicate that combining different data sources can improve prediction accuracy.

Chinnasamy et al. [7] addressed the forecasting of cardiovascular illnesses. The researchers compared and evaluated various ML algorithms in predicting cardiovascular illnesses. In addition, the study investigates the significance of feature selection, model optimization, and the use of large data in cardiovascular disease prediction. Overall, the article sheds light on ML algorithms that might be used to improve cardiovascular disease prediction and management.

Kiran et al. [8] investigated supervised ML algorithms in the identification and detection of cardiac attacks. This research compares the performance of various machine‐learning methods in detecting cardiac crises. The necessity of data preparation and feature selection in ML‐based heart attack diagnosis is also discussed. The supervised ML approaches have the potential to increase the accuracy and speed of heart attack detection, resulting in better patient outcomes. Jegedeesan et al. [9] described neural networks and other algorithms for the forecasting on cardiovascular diseases. The study's dataset contains a number of medical parameters. The authors compared the efficacy of various algorithms, including support vector machines, logistic regression etc., and concluded that neural networks provide the most accurate cardiovascular disease prediction. However, no experimental validation of the proposed model using actual data is included in the study. Ali et al. [10] analyzed the detection of the heart diseases by the supervised ML technique. Here several types of tools such as classification and prediction techniques in supervised ML tool to label the dataset from the different algorithm then the non‐weighted labelled dataset then loaded to categories the dataset and identifies the heart diseases for the categorized attributes. Using feature scoring and coefficient score the ranks are evaluated for maximizing the prediction of the heart disease.

## 3 PROPOSED METHODOLOGY

### 3.1 Random forest model

**FIGURE 1:**
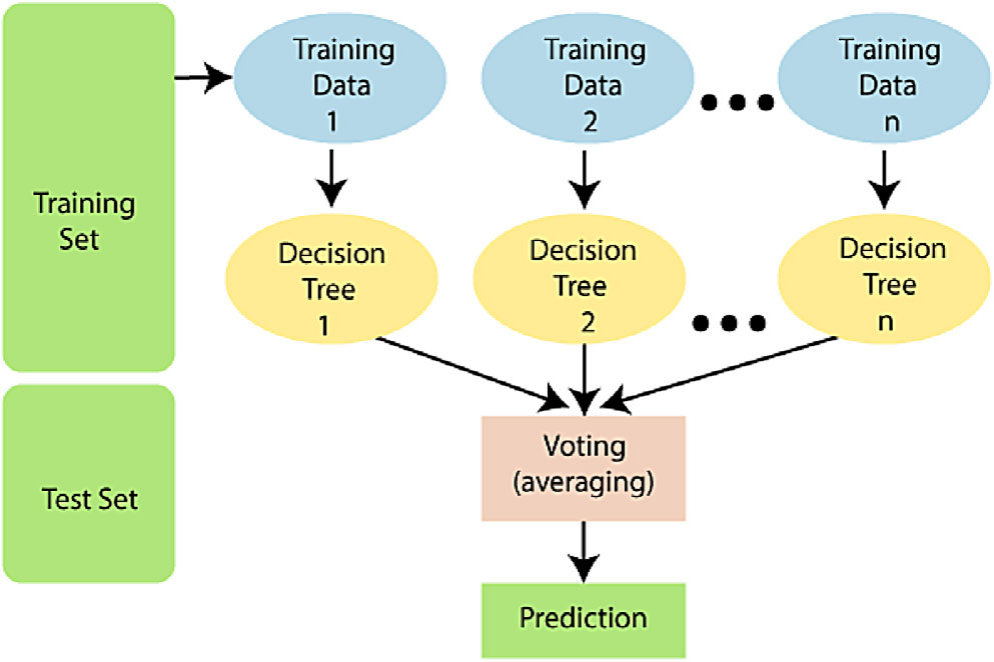
Random forest working process.

The ensemble learning technique known as random forest (RF) model is widely used for most supervised techniques. The method relies on a combination of several decision trees, each of which is trained using only a fraction of the input features and the training data as depicted in Figure 1. Each decision tree in a random forest is constructed in the same way that a decision tree would be but using a different selection of data and features. The model's generalization ability is enhanced, and overfitting is diminished, thanks to this randomness. The random forest algorithm makes a forecast mean the results of all (in regression issues) or by a simple majority (in classification problems). When working with noisy or missing data, this ensemble method can increase the model's accuracy and robustness.

Random forest can process data with missing values and accepts numeric and categorical input features. Important features for predictions can be identified with the help of the feature important scores provided. Accuracy is only a few of the metrics that may be used to assess a random forest's performance. Due to its ability to lower the model's variance and hence produce more consistent and dependable predictions, it is generally favoured over single‐decision trees. However, random forests may not do well on very big datasets and can be computationally intensity.

### 3.2 Pre‐processing the dataset

Pre‐processing the Data: To eliminate any inconsistencies or errors. This includes coping with missing values, outliers, and normalizing the data. Data pre‐processing is essential to ensure that the data can be utilized for model training.

Feature Extraction: The most pertinent features to the problem are chosen. This includes eradicating any unnecessary or redundant features. Feature selection is essential because it reduces the model's complexity and enhances its performance.

Selecting Model: Entails selecting the optimal algorithm for the given problem. This includes evaluating the effectiveness of various algorithms on the data set [11].

**FIGURE 2:**
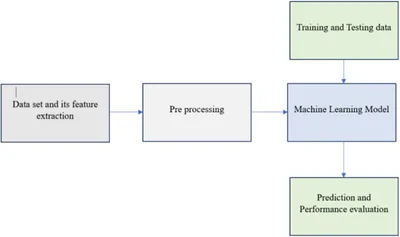
Process involved in predicting best result.

Evaluation of the Model: The final stage is to estimate chosen code from test data set. This involves comparing the model's predicted output to the actual output. In this research problem we have performed the Exploratory data analysis of various features of patient data set, that contains the attributes such as health parameters that involved for comparing disease affected with healthy people and unhealthy people. The process involved is shown in Figure 2.

### 3.3 Algorithm

Step 1: Each decision tree in the Random Forest model is given a collection of data points and features. Simply put, a *k*‐record data set is used to select *n* random records and *m* characteristics.
Step 2: A distinct decision tree is built for each sample.
Step 3: Each decision tree will produce a result.
Step 4: The Classification and Regression results are evaluated using Majority Voting or Averaging.

## 4 EXPERIMENTAL PROCEDURE AND DATASET ANALYSIS

**FIGURE 3:**
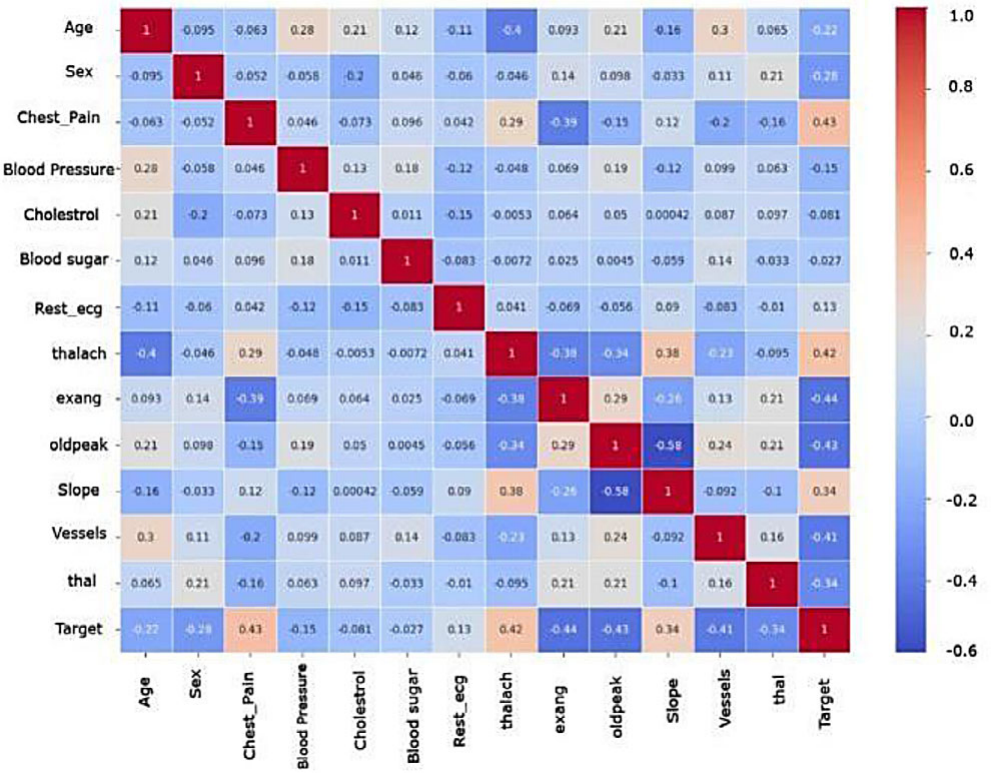
Correlation of patient's health with respect to all attributes.

Numerous features of the patients’ data are retrieved based on health criteria for 301 patients with mixed sex (male and female) and 14 attributes. Initially, we categorized the patients as male or female depending on their gender and health status, with 206 male and 96 female. There are 114 healthy male patients and 92 sick male patients. Healthy and unwell females are 24 and 74, respectively. The correlation of patient's health with respect to all attributes is given in Figure 3.

**FIGURE 4:**
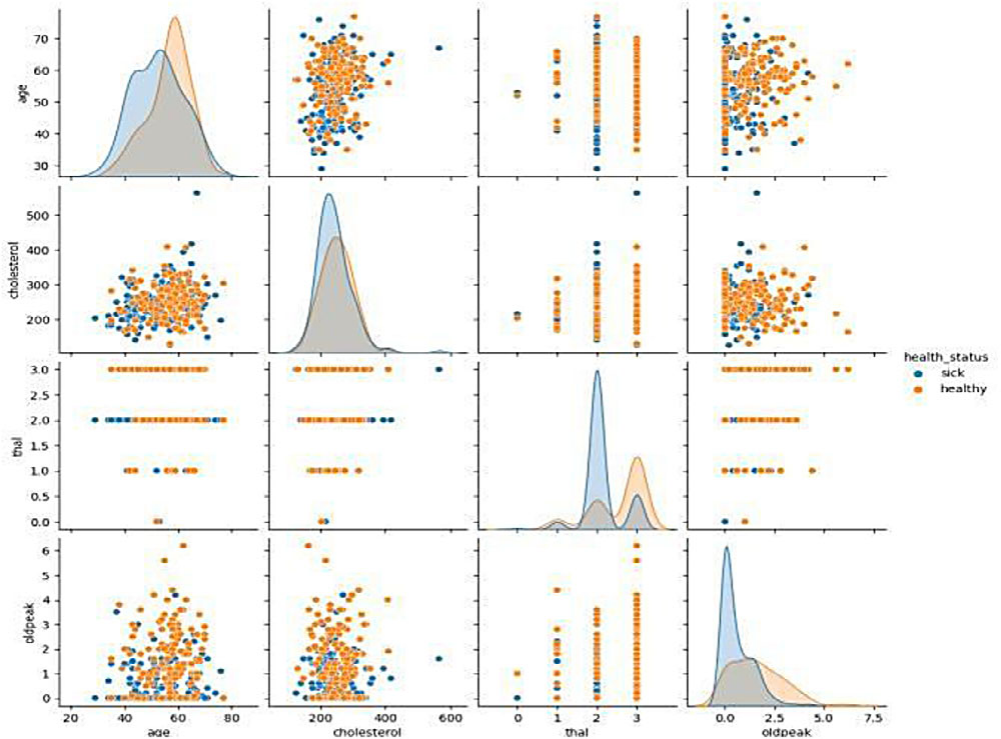
Comparison of all attributes.

Also compared with patients' health condition with all metrics and generated correlation plot, which gives the whole information of patient health concerning risk factor for heart attack. Similarly, all the health parameters are compared and plotted as given in Figure 4.

## 5 COMPARING MODEL AND BUILDING THE PREDICTIVE MODEL

**FIGURE 5:**
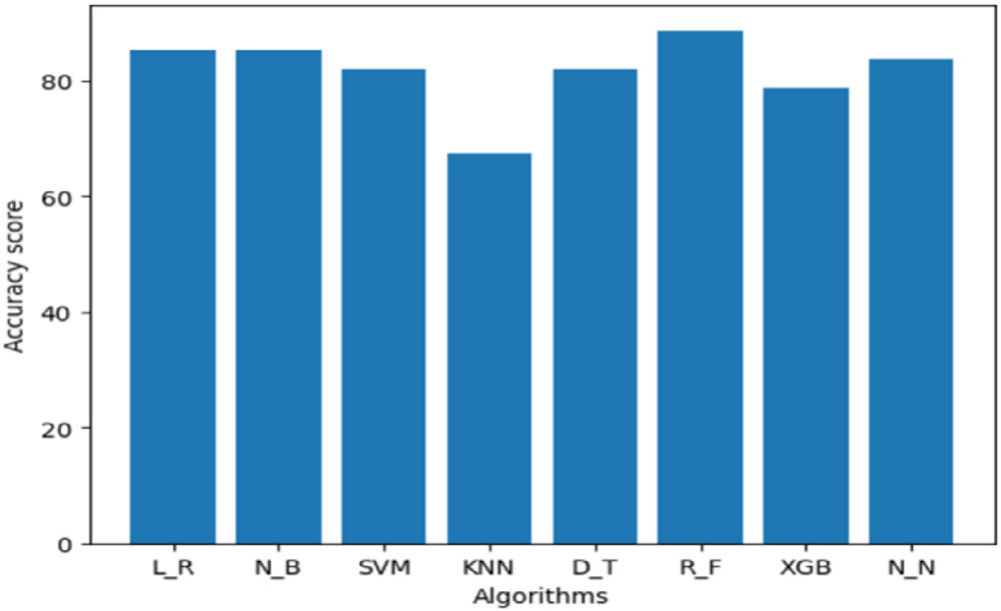
Comparison of various algorithms.

Seven different ML methods are used in the study problem. For the data set we trained, the Logistic Regression model achieved an accuracy of 85.25%. We obtained an accuracy score of 8525% in the Naive Bayes algorithm for this trained model, which is the same as the Logistic regression model. Similarly, we modelled the SVM, which performed a score of 81.97%. Although we tested other algorithms, such as the Decision Tree model, it achieved a score of around 81.97%, K‐nearest neighbour achieved 67.21%, Random Forest model reached 88.52%, XG‐boost scored approximately 78.69%, and Neural networks method achieved approximately 83.61%. Figure 5 shows the comparative analysis of various algorithms.

**FIGURE 6:**
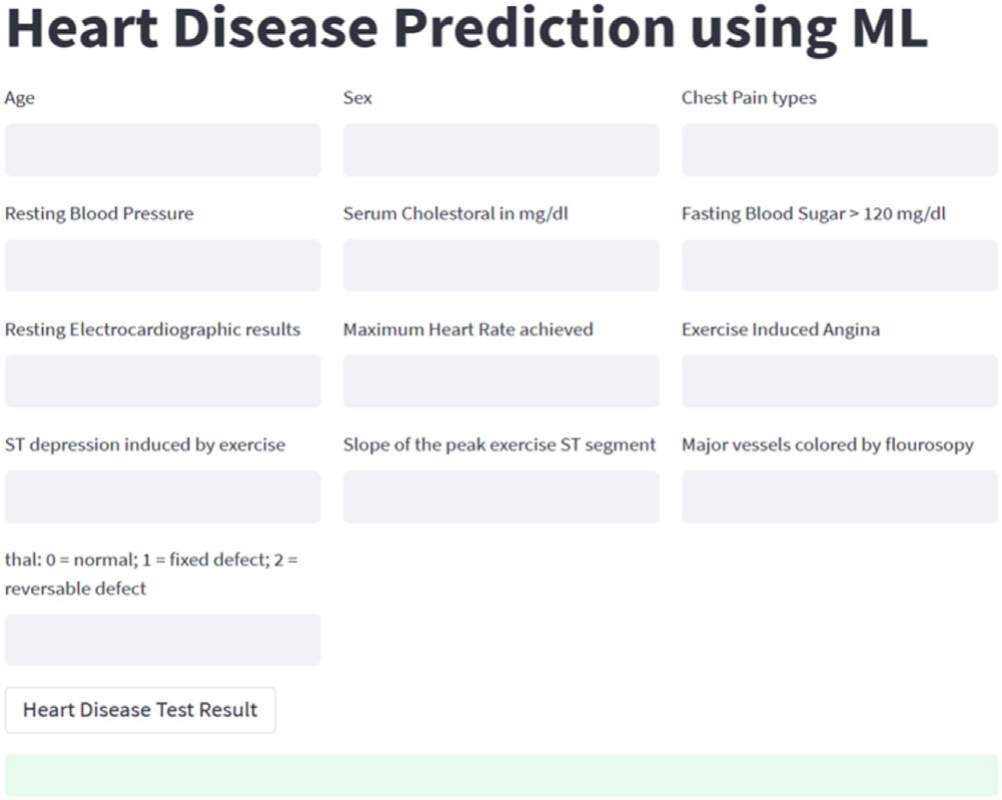
Web interface for parameters of heart disease.

When comparing all the algorithms used for training, it is found that the Random Forest approach gave the most probable outcome of 88.52% of all the models created. So, based on this algorithm, the final predictive model is constructed that achieves the highest accuracy in predicting the risk of patients with heart attacks based on the attributes of patients' medical data. We also developed a user interface for all the attributes, which can be used to check the patients' risk factor based on their health parameters, whether they have high risk or not. Figure 6 depicts the developed Web interface for parameters of heart disease.

### 5.1 Edge over other algorithms

Random Forest is a highly effective ML algorithm that is renowned for its high predicted accuracy, low overfitting, and feature importance. It outperforms singular decision trees and standard algorithms, making it suitable for noisy or complex datasets. Random Forest is able to estimate its own generalization error using out‐of‐bag samples, which simplifies model evaluation. It can handle missing data without requiring extensive data imputation or preparation, making decisions based on the available information for each sample. It is ready parallelization, making it appropriate for enormous datasets and distributed computing environments. Random Forest can be utilized for various ML tasks, including classification, regression, and unsupervised learning. It is less susceptible to outliers and can ‘average out’ the effect of extreme numbers due to its ensemble structure. Random Forest, as a non‐parametric method, makes no assertions about the distribution of the underlying data, making it suitable for a wide variety of data types and distributions. Its diverse ensemble of models contributes to its adaptability and generalization.

## 6 CONCLUSION

Using the Random Forest ML algorithm to improve heart attack prediction is a major advance in the direction of better assessing and managing cardiovascular health. This research demonstrates the immense potential of Random Forest as a method for predicting who will have a heart attack. Random Forest models have been shown to be effective in identifying high‐risk subjects and providing healthcare providers with the data they need to make informed decisions about prevention and intervention by analyzing large datasets containing a wide variety of demographic, clinical, and lifestyle variables. Random Forest is superior than other methods in assessing cardiac event prediction. Its outstanding potential to handle high‐dimensional data and missing values with ease would be extremely useful in practical healthcare applications. The feature relevance scores supplied by the Random Forest algorithm also work as a guidepost, leading doctors and nurses in the direction of the most crucial risk variables and facilitating the prioritization of therapies most likely to result in sizable reductions in cardiovascular disease mortality. One more significant advantage is that Random Forest models are easy to understand and use. These models help healthcare providers and people have more fruitful conversations about risks by clarifying the gravity of individual dangers. Patient compliance with treatment plans and behavioural modifications is increased when patients have a firm understanding of their unique risk profiles.

However, it is important to keep in mind that the performance of Random Forest models in forecasting heart attack risk is greatly dependent on the quantity and quality of the training data, as well as the careful selection of relevant features. Long‐term dedication to amassing complete and up‐to‐date datasets and refining the feature selection technique is necessary to guarantee the model's correctness, precision, and dependability. The Random Forest ML algorithm provides a very encouraging new approach to forecasting heart attack risk, which could herald a revolution in the diagnosis and treatment of cardiovascular illnesses. The use of AI in conjunction with data could improve risk assessment, lead to more precise health promotion initiatives, avert heart attacks, and save lives. It is important to note that ML models are not meant to replace the expertise of medical professionals; clinical judgment and individual attention to patients are still necessary for the effective management and prevention of heart disease. The continued synergy between data‐driven techniques and clinical competence bodes well for the future of cardiovascular health and the prevention of heart attacks in high‐risk groups.

## AUTHOR CONTRIBUTIONS

**Albert Alexander Stonier**: Wrting the original draft and reviewing; Supervision. **Rakesh Krishna Gorantla**: Software; Validation; Writing—original draft. **K Manoj**: Resources; Software; Visualization; Writing—review & editing.

## CONFLICT OF INTEREST STATEMENT

The authors declare no conflict of interest.

## Data Availability

The datasets analyzed during the current study are available from the corresponding author on reasonable request.
